# Thiolated Janus Silsesquioxane Tetrapod: New Precursors for Functional Materials

**DOI:** 10.3390/molecules27227680

**Published:** 2022-11-08

**Authors:** Mathilde Laird, Carole Carcel, Masafumi Unno, John R. Bartlett, Michel Wong Chi Man

**Affiliations:** 1 ICGM, Univ Montpellier, CNRS, ENSCM, 34293 Montpellier, France; 2 Department of Chemistry and Chemical Biology, Graduate School of Science and Technology, Gunma University, Kiryu 376-8515, Japan; 3 School of Science, Western Sydney University, Locked Bag 1797, Penrith, NSW 2751, Australia

**Keywords:** T_4_ Janus silsesquioxane, Janus tetrapod, bifunctional tetrapod

## Abstract

Herein, we report synthetic strategies for the development of a bifunctional Janus T_4_ tetrapod (Janus ring), in which the orthogonal silsesquioxane and organic faces are independently functionalized. An all-*cis* T_4_ tetrasilanolate was functionalized to introduce thiol moieties on the silsesquioxane face and naphthyl groups on the organic face to introduce luminescent and self-organization properties. The stepwise synthesis conditions required to prepare such perfectly defined oligomers via a suite of well-defined intermediates and to avoid polymerization or reactions over all eight positions of the tetrapod are explored via ^29^Si, ^13^C and ^1^H NMR, FTIR and TOF-ESI mass spectroscopy. To the best of our knowledge, this is one of the few reports of Janus T_4_ tetrapods, with different functional groups located on both faces of the molecule, thus expanding the potential range of applications for these versatile precursors.

## 1. Introduction

Silsesquioxane compounds, defined by the general formula (RSiO_1.5_)_n_ where R is an organic group, extend from simple molecular systems to complex material architectures [1]. Random networks [2] together with well-defined molecules [3] can be obtained from the hydrolysis and condensation of trialkoxy- or trichloro-silanes. In 1965, Brown and Vogt first described a range of well-defined oligomeric silsesquioxanes, together with some of their precursors, including the T_4_ tetrasilanols [4]. T_4_ tetrasilanols/silanolates have been demonstrated to be versatile precursors for producing a variety of oligomeric silsesquioxanes such as cages [4,5], Janus cages [6,7,8,9], ladder silsesquioxanes [9,10] and in particular silsesquioxane tetrapods [11].

**Figure 1:**
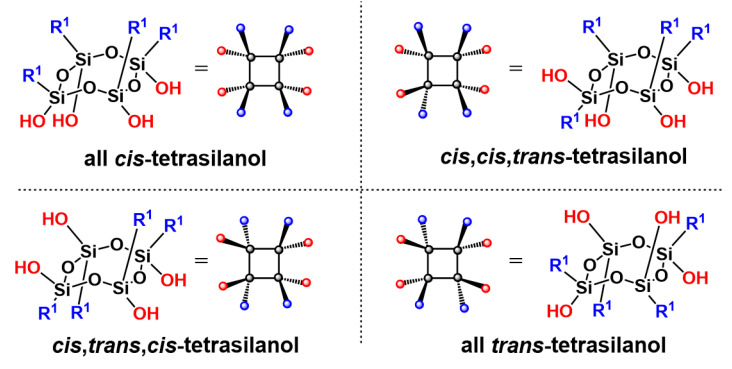
T_4_ tetrasilanol isomers.

As the Si-O bond is typically around 1.6 Å in length [11,12], a particular feature of oligomeric silsesquioxanes is that the organic groups are in close proximity to one another, with separations of less than a nanometer. In the case of cage silsesquioxanes, which are the oligomeric silsesquioxanes most widely reported in the literature [13,14] and for which potential applications have been explored [13,15,16], improved responses due to the proximity effect have been demonstrated in catalysis [17], optics [18,19,20] and sensing [21,22]. On the contrary, the design of functional tetrapods is still in its infancy, thus restricting potential applications of such structures. Although cyclotetrasiloxanes with various substituents have been reported [23,24], expanding the scope of tetrapod silsesquioxanes has been significantly limited by both the reduced possibility of functionalization of T_4_ tetrasilanolates and by the difficulty of obtaining T_4_ tetrasilanols with well-defined structures. In particular, T_4_ silanols and silanolates can adopt four isomeric forms: the all-*cis*, *cis*,*cis*,*trans*, *cis*,*trans*,*cis*, and the all-*trans* T_4_ isomers (Figure 1) [25,26,27,28,29]. Of these, all-*cis* isomers possess two distinct, well-defined faces (Janus molecule). Potential applications for these orthogonally functionalizable molecules include the design and synthesis of well-defined cage silsesquioxanes, catalysts, surface functionalization, protective coating, etc. [23]. Although tetrasilanolates bearing organic ligands have been previously described, including alkyl- [27], methyl- [30], phenyl- [31], vinyl- [32], halogenophenyl- [33] and styryl-functionalized T_4_ tetrasilanolate [33], their poor solubility is one of the major obstacles impeding their post-synthesis functionalization. The T_4_ tetrasilanols exhibiting the highest solubility are mainly obtained from the hydrolysis of T_4_ tetrasilanolates [31] or hydrolysis–condensation of trichlorosilanes [25,34]. However, their synthesis is susceptible to multiple side reactions, such as isomerization or polymerization, which significantly reduce the availability of T_4_ tetrasilanols described as pure isomers [35]. To the best of our knowledge, the condensation reaction of a chlorosilane on T_4_ tetrasilanolate, under well-controlled reaction conditions, is the only method reported to afford T_4_ silsesquioxanes [10,25,36,37] in a controlled fashion. Janus T_4_ tetrapods, i.e., with different organic functions on opposite faces, are thus obtained by the condensation of a commercially available chlorosilane on a T_4_ tetrasilanolate [11].

**Figure 2:**
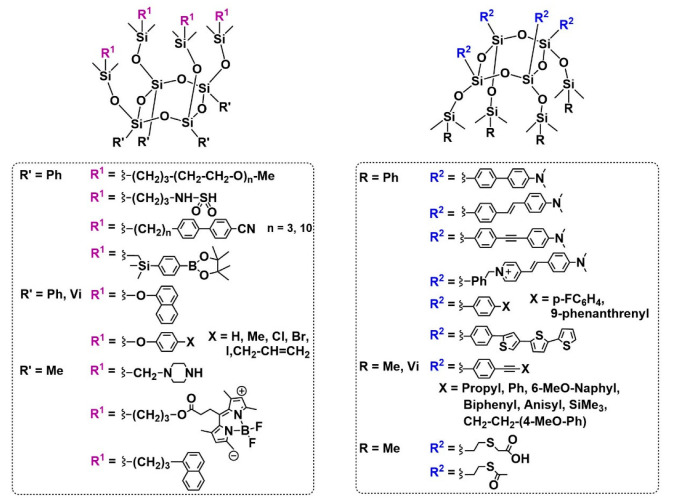
Existing functional Janus tetrapod silsesquioxanes [11,23,24,37,38,39,40,41,42,43,44,45,46,47,48,49,50,51].

Using this functionalization approach, several functional Janus tetrapod silsesquioxanes have now been reported, together with details of their synthesis in some cases (Figure 2) [11,23,24,37,38,39,40,41,42,43,44,45,46,47,48,49,50,51]. To the best of our knowledge, these T_4_ compounds have all been functionalized on the silanol/silanolate face of the precursor molecule via condensation reactions with commercially available chlorosilanes, to yield a Janus tetrapod silsesquioxane. However, post-functionalization can be realized either on the Si-O-SiMe_2_R face, mainly by hydrosilylation [37,38,39,41,42,43,50,51] or by the Piers–Rubinsztajn reaction [23,24]; or on the Si-C face using a wide range of standard reactions including Suzuki [44], Sonogashira [11,45] and Heck coupling reactions [11,45,47], etc. The resulting tetrapodal compounds have a range of applications, including self-organizing systems [37,38,39]; chromophores [44,45,46,47,48] and photoexcitation [44]; flame-retardant and water-repelling applications [40]; liquid crystals [11,37,38,41]; or as a fluorescent dye [43]. In some of the applications, the inherent geometrical constraints associated with the T_4_ cycles conferred interesting properties on the tetrapods. These include the formation of tetrapodal excimers [44,48] from organic monomers that do not exhibit such properties. However, it is important to note that, in all of these cases, only one face of the Janus tetrapod silsesquioxane confers these properties on the molecule.

Herein, we report the development of a new bifunctional Janus T_4_ tetrapod, in which the orthogonal silsesquioxane and organic faces are independently functionalized (R^1^ and R^2^, respectively, in Figure 2). All-*cis* T_4_ tetrasilanolates were functionalized to introduce (a) four thiol moieties on the silsesquioxane Si-C face (R^2^); and (b) four naphthyl groups on the organic Si-O-Si(Me)_2_-R face (R^1^). The design of the stepwise synthesis employed, which enables well-defined intermediates and oligomers to be obtained, is explored, extending the scope of potential applications for this interesting family of molecular precursors. Naphthyl- and thiol-functionalized groups were chosen for this proof-of-concept study due to the luminescence and self-organization properties of the former, and the potential for modification of the latter via thiol-ene click reactions or binding to metal nanoparticles such as Au.

## 2. Results and Discussion

In our work, a T_4_ silanolate (tetravinylcyclotetrasilanolate, potassium salt [32]) was chosen as the precursor from which to prepare the Janus T_4_ tetrapod, because of its more facile synthesis and relatively good stability compared to T_4_ tetrasilanols. The approach involved the use of an all-*cis* tetravinylcyclotetrasilanolate, to take advantage of the many different reactions available for the functionalization of the vinyl C=C bond, including metathesis, Heck coupling, hydrosilylation and thiol-ene click reaction. Further functionalization of the silanolate precursor, by the condensation of a chlorosilane on the silanolate face, was then envisaged. However, as previously mentioned, the variety of commercially available chlorosilanes is limited. The organic groups are generally restricted to moieties such as aliphatics (methyl, *t*-Bu, octadecyl), C_6_F_5_, etc., which are difficult to post-functionalize. Chlorosilanes with nucleophilic substituents (chloromethyl, cyanopropyl) or phenyl derivatives are also available, but these can induce unwanted side reactions during different post-functionalization steps. Chlorodimethylsilane and vinyl derivatives were also discounted, as orthogonal functions are required between the silanolate and the Si-C face to prevent polymerization or reaction over all eight positions of the tetrapod. Accordingly, a tailor-made chlorosilane was synthesized from an ethoxysilane precursor, as described below, to avoid the issues of pronounced reactivity, sensitivity to air and water as well as limited purification options associated with direct modification of a commercial chlorosilane. 

### 2.1. Synthesis of Ethoxydimethyl(2-naphthylethyl)silane

Initial scoping studies employed a commercially-available dimethylvinylethoxysilane, ViMe_2_SiOEt, which was reacted with 2-bromonaphthalene to obtain a conjugated ethoxysilane by Heck coupling [52,53]. However, both *E*- and *Z*-stereoisomers were identified in the crude reaction product, which was obtained in low yield (14%). Subsequent attempts to purify the mixture and separate the isomers by column chromatography were unsuccessful. 

**Figure 3:**
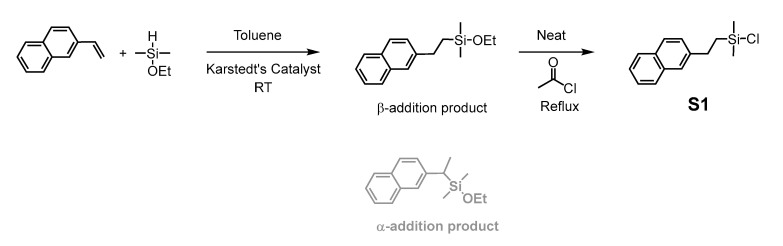
Synthesis of chlorodimethyl(2-naphthylethyl)silane (**S1**). The by-product resulting from α-addition is shown in gray (removed by flash chromatography prior to reaction with acetyl chloride).

The hydrosilylation reaction [54,55] was thus performed with ethoxydimethylsilane and 2-vinylnaphthalene using Karstedt’s catalyst (Figure 3). The ^1^H NMR spectrum of the crude product revealed the absence of signals associated with the vinylic protons of the vinylnaphthalene, indicating completion of the reaction. Although the Karstedt’s catalyst generally leads to the product of β-addition, both regioisomers could be identified in the crude reaction product in a ratio of 25/75 α- to β-addition products. Following the purification of the crude mixture by flash column chromatography to isolate the pure β-addition product (recovered with 40% yield), the corresponding ^1^H NMR spectrum (Figure S1) exhibited CH_2_ signals at 2.84 and 1.07 ppm arising from the hydrosilylation of the double bond. The ethoxy signals are also present at 3.72 and 1.20 ppm and the methyl groups bonded to the silicon are attributed at 0.16 ppm. No impurities were evident in the spectrum. In addition, the integrations are consistent with the expected product. Similarly, all signals observed in ^13^C NMR are consistent with the formation of the β-addition product (Figure S2). In the ^29^Si NMR spectrum (Figure S3), a single signal is observed at 16.70 ppm, consistent with that usually observed for compounds similar to ethoxydimethyl(alkyl)silane [56,57]. The product was confirmed by ESI-MS with a measured mass of 213.1 (M after OEt loss)^+^ for an expected 213.1 m/z. The FTIR spectrum of the product also exhibits the characteristic band at 3055 cm^−1^ arising from ν(C_Napht_-H), together with the antisymmetric and symmetric ν(Si-O-C) modes at 1077 and 942 cm^−1^, respectively [58,59]. 

Triethoxysilanes, when involved in condensation reaction with silanolates, are not as reactive as chlorosilanes and can release basic ethanolates, which can interfere with the reaction. Indeed, attempts to directly graft the ethoxydimethyl(2-naphthylethyl)silane onto the T_4_ silanolate were unsuccessful. In contrast, chlorosilanes, which form neutral chloride salts as a byproduct during condensation, avoid this side reaction. Consequently, the ethoxydimethyl(2-naphthylethyl)silane was chlorinated using acetyl chloride as a chlorinating agent, as previously described [60] (Figure 3), yielding **S1**. The ^1^H NMR spectrum (Figure S4) demonstrates the disappearance of the ethoxy-group signals, as well as a shift of the dimethyl signals toward the lower field due to the proximity of the more electronegative chlorine atom. Similarly, the CH_2_ signals are displaced downfield to 2.97 and 1.33 ppm. No significant impurities can be observed in the spectrum. The ^13^C NMR spectrum is also consistent with the chlorination, as evidenced by the disappearance of the ethoxy signals around 18 and 58 ppm and by the shift of the dimethyl signal from −1.95 to 1.77 ppm (Figure S5). Moreover, in ^29^Si NMR (Figure S6), the silicon signal is shifted to 31.61 ppm, characteristic of the chlorosilane [56]. The ESI-MS data were also consistent with the target compound, with a 248.1 m/z corresponding to the expected value for **S1**.

### 2.2. Synthesis of Bifunctional T_4_ Janus Tetrapod

**Figure 4:**
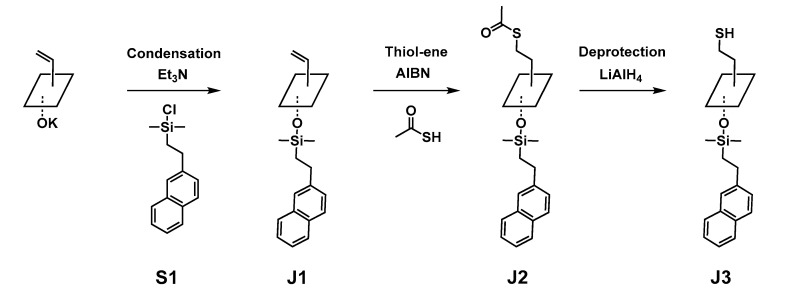
Synthetic route to the bifunctional Janus T4 tetrapod (note that 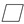 refers to the Si4O4 ring, as il-lustrated in Figure 1).

The chlorosilane described above was subsequently grafted onto the all-*cis* T_4_ tetrasilanolate [32] via condensation. This significantly enhances the solubility of the resulting T_4_ in common solvents such as chloroform, dichloromethane, tetrahydrofuran and toluene, thus facilitating functionalization on the Si-C face (Figure 4). The target Janus T_4_ tetrapod was then obtained via a three-step reaction: (1) condensation to graft the naphthyl chlorosilane onto the silanolates; (2) thiol-ene click reaction with thioacetic acid at the vinyl site to introduce a protected thiol moiety; and (3) deprotection of the latter to release the thiol function. Other recent reports of the synthesis of functional Janus tetrapods with thioacetate [49] or naphthalene groups [24,50] have yielded products which either bear functional groups on only one face; have vinyl moieties on both faces (thus preventing a selective functionalization of one face of the cycle); or were not further functionalized. In contrast, our approach enables the orthogonal faces to be independently functionalized.

**Figure 5:**
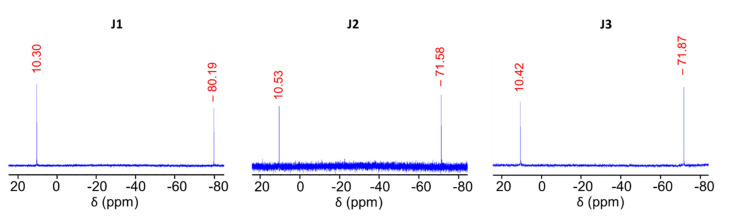
^29^Si NMR spectra of **J1**, **J2** and **J3** (CDCl_3_).

Firstly, the all-*cis* T_4_ vinyl silanolate was reacted with the chlorosilane **S1** in the presence of triethylamine as an HCl scavenger to avoid isomerization of the T_4_ structure [35]. A small excess of **S1** was used to ensure complete substitution on the four silanolate sites. The resulting oil was purified via flash column chromatography to remove the main impurity, namely, the disiloxane formed by the hydrolysis of the excess **S1**. The ^1^H NMR spectrum of the pure **J1** (Figure S7) shows signals arising from both naphthyl (7.33 to 7.80 ppm) and vinyl protons (5.99 to 6.01 ppm). In addition, the signals from CH_2_ associated with the chlorosilane are shifted upfield to 1.10 (**CH_2_**-Si) and 2.86 ppm (**CH_2_**-CH_2_-Si) due to the substitution of the chlorine by an oxygen atom. The dimethyl groups of the silane experience a similar effect and are seen at 0.25 ppm. In addition, the small signals observed on the base of the main signals are due to the presence of small quantities of some isomers, which could not be separated by column chromatography. In the ^29^Si NMR spectrum of **J1** (Figure 5, left), two signals can be seen at 10.30 and −80.19 ppm. The former corresponds to the dimethyl(2-naphthylethyl)silane linked to an oxygen atom, while the latter is consistent with the signals typically obtained for T^3^ silsesquioxane species linked to an unsaturated organic group. Both chemical shifts are consistent with the literature [37]. It is noteworthy that no additional signals associated with the chlorosilane or its hydrolysis/self-condensation products are observed. In addition, FTIR (Figure S8) and mass spectrometry corroborate the results obtained by NMR. In particular, the ESI-MS exhibits a strong peak at 1201.41 m/z (M+H)^+^ for an expected m/z of 1201.40. In the FTIR spectrum, the naphthyl group can be observed through the C-C and C-H stretching modes at 1600 and 3056 cm^−1^, respectively [59]. The CH in-plane bending mode of the alkene [61] can be seen at 1366 cm^−1^ and the Si-Me stretching mode of the dimethylsilane [37] at 841 cm^−1^. Furthermore, the Si-O-Si stretching mode of the silsesquioxane is observed at 1043 cm^−1^, which is higher than that of the T_4_ tetrasilanolate (952 cm^−1^). This shift is consistent with a more constrained T_4_ ring than that of the unfunctionalized tetrasilanolate. The sharpness of the Si-O-Si peak also confirms that the product obtained is not a polymer, but a well-defined cyclic oligomer, consistent with the formation of **J1**.

The grafting of the chlorosilane onto the T_4_ tetrasilanolate significantly increases its solubility, thus facilitating the functionalization of the T_4_ on the opposite Si-C face. Accordingly, **J1** was subjected to a thiol-ene click reaction with thioacetic acid to introduce a thiol function in its protected form (**J2**, Figure 4), following a procedure previously developed by our group [62]. The ^1^H NMR spectrum of the crude product indicated the completion of the reaction, together with the presence of impurities. After purification by silica column chromatography, the ^1^H NMR spectrum of **J2** (Figure S9) was consistent with the complete functionalization of the four vinyl groups of **J1**, as shown by the disappearance of the vinyl signal at 6.00 ppm. A new CH_2_ signal at 2.98 ppm, which corresponds to the expected chemical shift of the CH_2_ group in the α-position of the sulfur atom, was observed. Indeed, the electronegativity of the sulfur atom leads to lower field shifts. In addition, the signal at 1.04 ppm appears as a complex multiplet after grafting. Due to the proximity of the silicon atoms of the T_4_ ring, the two CH_2_ protons in the α-position of the silicon atoms on each face of the ring can appear together and thus be nearly superimposed. The signal corresponding to the thioacetate is observed at 2.29 ppm. In addition, it should be noted that column chromatography enables some of the isomers generated during the condensation step to be removed. Indeed, the signal associated with the dimethylsilane is very sharp and the signals near the base of the main signal, observed in the spectrum of **J1** (Figure S7), were not evident in Figure S9. The changes in polarity between the naphthylvinyl T_4_ silsesquioxane and naphthylthioacetate T_4_ silsesquioxane may have facilitated the improved separation. The ^29^Si NMR spectrum also exhibits a major change in the silicon chemical shifts (Figure 5, middle). In particular, the silicon linked to the organic group of the T^3^ silsesquioxane species shifts from −80.19 ppm in the case of the vinyl substituent (unsaturated) to −71.58 ppm in the case of the ethylthioacetate substituent (saturated). This result is consistent with the expected chemical shift of the T^3^ silsesquioxane linked to a saturated organic group, which is expected at around −70 ppm [32]. Furthermore, the FTIR spectrum (Figure S8) is consistent with the NMR data. The thioacetate C=O stretching mode appears at 1687 cm^−1^ and the (C=O)-S stretching mode at 624 cm^−1^ [63]. The characteristic naphthyl bands are also visible, including the C-C and C-H stretching modes at 1600 and 3055 cm^−1^, respectively [59]. The Si-Me stretching mode of the dimethylsilane [37] is still visible at 842 cm^−1^. The Si-O-Si stretching mode of the silsesquioxane [64] is observed at 1053 cm^−1^ as a sharp peak, consistent with a well-defined T^3^ silicon species such as **J2**. The ESI-MS data also confirm the formation of **J2**, with a peak observed at 1527.42 m/z (M+Na)^+^ for an expected value of 1527.38 m/z. 

To release the thiol function, the deprotection of the **J2** thioacetate is required. Due to the possibility that the released thiol would oxidize, all reactions were carried out in solvent outgassed by freeze–pump–thaw cycles. As silsesquioxanes are generally sensitive to basic and nucleophilic media, the deprotection was first performed under acidic conditions with 35% hydrochloric acid solution [65] or with in situ acid generated from acetyl chloride [66]. In both cases, the thiol was successfully deprotected, but isomerization and/or polymerization was observed via the broadening of the NMR signals. Additional trials involved the use of potassium carbonate [67] as a weak base and weak nucleophile, but no reaction occurred even after three days. Finally, the successful deprotection was achieved under reducing conditions using LiAlH_4_. The crude **J3** product obtained after completion of the reaction was purified by flash column chromatography to remove small quantities of isomeric impurities. The ^1^H NMR spectrum of **J3** (Figure S10) illustrates the disappearance of the acetate signal associated with the thioacetate at 2.29 ppm, together with the sharp dimethyl signal at 0.17 ppm expected for the pure compound. In addition, signals arising from the CH_2_ in the α-position of the silicon atoms are present at 1.03 ppm, while those in the α-position of the thiol at 2.75 ppm (similar shift to that of the thioacetate) and the α-position of the naphthyl group at 2.62 ppm are also observed. Similarly, the ^29^Si NMR spectrum of **J3** (Figure 5**,** right) shows two signals: one at 10.42 ppm corresponding to the dimethylsilane, and a second at −71.87 ppm corresponding to the T_4_ cycle (T^3^ silsesquioxane species linked to saturated moieties). The sharp signals observed in ^29^Si NMR confirm that the degradation and isomerization by-products were successfully removed. In addition, the FTIR spectrum (Figure S8) shows the disappearance of the thioacetate C=O mode (1687 cm^−1^) [63]. The weak thiol S-H stretching mode [68] appears at 2570 cm^−1^, confirming successful deprotection. The characteristic bands of naphthyl are also observed, with the C-C and C-H stretching modes observed at 1600 and 3051 cm^−1^, respectively [59]. A sharp Si-O-Si stretching band of the silsesquioxane T^3^ species is still seen at 1047 cm^−1^, suggesting that the cyclic system remained intact following the treatment with LiAlH_4_, although the peaks appear slightly broader in the latter case. The ESI-MS data were also consistent with the formation of **J3**, with a peak observed at 1354.38 m/z (M+NH_4_)^+^ (expected value 1354.39 m/z).

**Figure 6:**
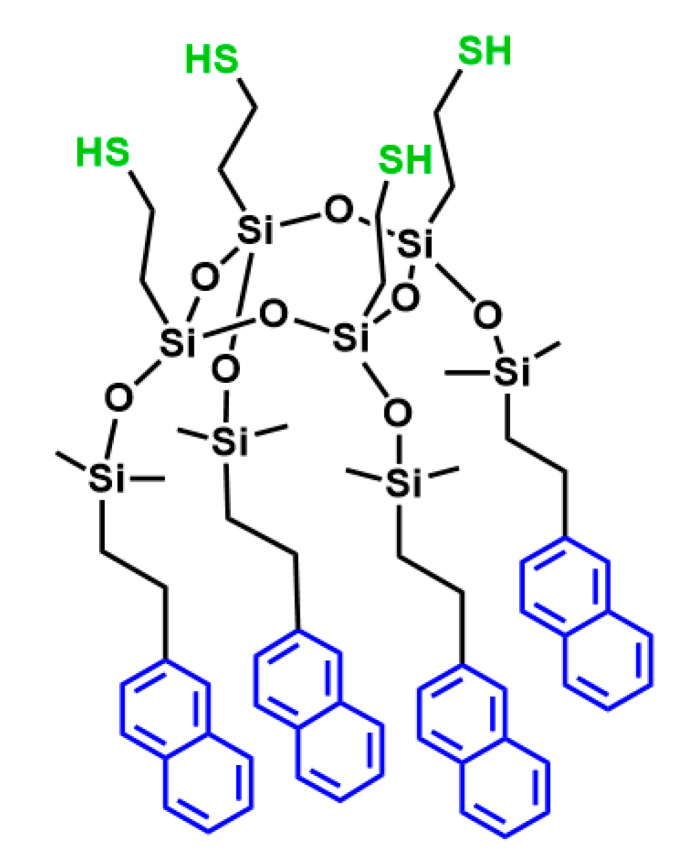
Janus T_4_ tetrapod in an all-*cis* configuration (**J3**).

These data confirm the successful synthesis of the target Janus T_4_ tetrapod in an all-*cis* configuration, as shown in Figure 6. This constitutes one of the few reports of the synthesis of such bifunctional compounds and is, to the best of our knowledge, the first report of a Janus T_4_ silsesquioxane bearing reactive thiol ligands on one face of the molecule. As demonstrated in previous reports, such ligands attached to both ring and cage silsesquioxanes can be readily post-functionalized using standard reactions such as thiol-ene click chemistry [62]. Such strategies involving **J3** will be explored in a future study.

## 3. Materials and Methods

### 3.1. Chemicals

All-*cis* T_4_ vinyl silanolate (tetravinylcyclotetrasilanolate, potassium salt) was prepared as described previously [32]. Azobisisobutyronitrile (AIBN) was recrystallized prior to use. 2-vinylnaphthalene and ethoxydimethylsilane were purchased from Alfa Aesar; Karstedt’s catalyst and triethylamine from Sigma Aldrich; and thioacetic acid and acetyl chloride from Acros. Lithium aluminum hydride was provided by TCI. All chemicals were used without any purifications. Toluene and THF were obtained from VWR and dried prior to use. 

The products were purified by flash chromatography in a Buchi Reveleris X2 flash chromatography system, equipped with a silica column, evaporative light-scattering detection (ELSD) and 254-nm light source, using a mixture of dichloromethane (DCM)/cyclohexane (VWR, technical grade) as eluents. 

### 3.2. Synthesis and Purification Methods

#### 3.2.1. Synthesis of Ethoxydimethyl(2-naphthylethyl)silane 

In a rotaflo^®^ Schlenk flask flamed-dried three times, 2-vinylnaphthalene (39 mmol, 6.0 g, 1 eq) was dissolved in toluene (20 mL) under an argon atmosphere. Ethoxydimethylsilane (58 mmol, 8.0 mL, 1.5 eq) was then added. The vessel was placed in a water bath to dissipate the heat generated by the highly exothermic reaction and avoid evaporation of the volatile ethoxydimethylsilane. Karstedt’s catalyst (2% Pt in xylene, 0.195 mmol, 2.2 mL, 0.5 mol% Pt with respect to vinylnaphthalene) was added dropwise. The completion of the reaction was verified by ^1^H NMR after 10 min. The solvent and excess silane were evaporated under reduced pressure after 1 h of reaction. Finally, the crude product was purified by flash column chromatography with a cyclohexane/DCM gradient up to 80/20. After separation and solvent evaporation, the product was recovered as a colorless oil. **Yield:** 40% (C_16_H_22_OSi, 4.0 g, 15.5 mmol) **^1^H NMR (400 MHz, CDCl_3_, δ, ppm):** 7.77 (m, 3H, **H_Napht_**), 7.64 (s, 1H, **H_Napht_**), 7.40 (m, 3H, **H_Napht_**), 3.71 (q, 2H, J = 17.5 Hz, O-**CH_2_**-CH_3_), 2.84 (t, 2H, J = 8.8 Hz, Napht-**CH_2_**-CH_2_-Si), 1.22 (t, 3H, J = 17.5 Hz, O-CH_2_-**CH_3_**), 1.07 (t, 2H, J = 8.8 Hz, Napht-CH_2_-**CH_2_**-Si), 0.16 (s, 6H, Si**Me_2_**) **^13^C NMR (100 MHz, CDCl_3_, δ, ppm):** 142.50 (**C_Napht_**), 133.77 (**C_Napht_**), 131.99 (**C_Napht_**), 127.89 (**C_Napht_**), 127.65 (**C_Napht_**), 127.47 (**C_Napht_**), 127.08 (**C_Napht_**), 125.89 (**C_Napht_**), 125.48 (**C_Napht_**), 125.05 (**C_Napht_**), 58.40 (O-**CH_2_**-CH_3_), 29.55 (Napht-**CH_2_**-CH_2_-Si), 18.67 and 18.31 (O-CH_2_-**CH_3_** and Napht-CH_2_-**CH_2_**-Si), −1.95 (Si**Me_2_**) **^29^Si NMR (80 MHz, CDCl_3_, δ, ppm):** 16.70 **IR (ν, cm^−1^):** 3055 (C_Napht_-H), 1598 (C_Napht_-C_Napht_), 1077 (Si-O-C), 943 (Si-O-C), 853 (Si-Me) **ESI-MS (M–ethoxy):** obtained 213.1 m/z, expected 213.1 m/z.

#### 3.2.2. Synthesis of Chlorodimethyl(2-naphthylethyl)silane (**S1**) 

In a flame-dried two-neck flask, ethoxydimethyl(2-naphthylethyl)silane (3.1 mmol, 795 mg) was added under an argon atmosphere. The ethoxysilane was mixed with acetyl chloride (2.4 mL, 10-fold excess). Finally, the reaction, held in neat conditions, was heated overnight to reflux under argon (80 °C). The excess acetyl chloride and volatile by-product (ethyl acetate) were evaporated under vacuum. The yellow oil product was handled at all times under argon and stored under an inert atmosphere in a refrigerator. **Yield:** quantitative (C_14_H_17_ClSi, ~746 mg, 3.0 mmol) **^1^H NMR (400 MHz, CDCl_3_, δ, ppm):** 7.82 (m, 3H, **H_Napht_**), 7.68 (s, 1H, **H_Napht_**), 7.48 (quin, 3H, J = 7.5 Hz, **H_Napht_**), 7.40 (d, 1H, J = 8.1 Hz, **H_Napht_**), 2.96 (t, 2H, J = 8.4 Hz, Napht-**CH_2_**-CH_2_-Si), 1.33 (t, 2H, J = 8.4 Hz, Napht-CH_2_-**CH_2_**-Si), 0.47 (s, 6H, Si**Me_2_**) **^13^C NMR (100 MHz, CDCl_3_, δ, ppm):** 141.33(**C_Napht_**), 133.73 (**C_Napht_**), 132.09 (**C_Napht_**), 128.08 (**C_Napht_**), 127.69 (**C_Napht_**), 127.50 (**C_Napht_**), 126.94 (**C_Napht_**), 126.03 (**C_Napht_**), 125.74 (**C_Napht_**), 125.25 (**C_Napht_**), 29.33 (Napht-**CH_2_**-CH_2_-Si), 20.81 (Napht-CH_2_-**CH_2_**-Si), 1.17 (Si**Me_2_**) **^29^Si NMR (80 MHz, CDCl_3_, δ, ppm)**: 31.61 **IR (ν, cm^−1^):** Not performed due to the corrosive nature of the product **ESI-MS** (in CDCl_3_) **(M–Cl)**: obtained 213.11 m/z expected 213.1 m/z **(M):** obtained 248.1 m/z expected 248.1 m/z.

#### 3.2.3. Synthesis of Tetrakis(dimethyl(2-naphthylethyl)silyloxy)tetravinylcyclotetrasiloxane (**J1**)

In a flame-dried Schlenk flask, T_4_ tetravinylcyclotetrasilanolate (0.87 mmol, 440 mg, 1 eq) was suspended in dry THF (4.4 mL) under an inert atmosphere. Distilled triethylamine (3.87 mmol, 540 µL, 4.4 eq) was then added, followed by the dropwise addition of the chlorodimethyl(2-naphthylethyl)silane (3.83 mmol, 955 mg, 4.4 eq). During the addition, a large quantity of salts was produced. The reaction mixture was held overnight at 25 °C. After reaction completion, the crude product was evaporated, dissolved in pentane to precipitate the triethylammonium salts formed and filtered on a celite^®^ pad. The evaporated filtrate was then purified by silica column chromatography with a cyclohexane/DCM gradient from 90/10 to 70/30 (by volume). Finally, the solvent was evaporated, and the pure **J1** product was recovered as a colorless oil. **Yield:** 63 % (C_64_H_80_O_8_Si_8_, 656.7 mg, 0.55 mmol) **^1^H NMR (400 MHz, CDCl_3_, δ, ppm):** 7.80 (m, 1H, **H_Napht_**), 7.73 (m, 2H, **H_Napht_**), 7.61 (s, 1H, **H_Napht_**), 7.44 (m, 2H, **H_Napht_**), 7.33 (dd, 1H, J_1_ = 8.5 Hz, J_2_ = 1.5 Hz, **H_Napht_**), 5.99 (m, 3H, **H_vinyl_**), 2.86 (t, 2H, J = 8.5 Hz, Napht-**CH_2_**-CH_2_-Si), 1.10 (t, 2H, J = 8.5 Hz, Napht-CH_2_-**CH_2_**-Si), 0.25 (s, 6H, Si**Me_2_**) **^13^C NMR (100 MHz, CDCl_3_, δ, ppm)**: 142.52 (**C_Napht_**), 135.20 (**CH_2_**=CH-Si), 133.73 (**C_Napht_**), 132.07 (**C_Napht_**), 131.94 (CH_2_=**CH**-Si), 127.82 (**C_Napht_**), 127.62 (**C_Napht_**), 127.46 (**C_Napht_**), 127.11 (**C_Napht_**), 125.79 (**C_Napht_**), 125.47 (**C_Napht_**), 124.95 (**C_Napht_**), 29.44 (Napht-**CH_2_**-CH_2_-Si), 19.93 (Napht-CH_2_-**CH_2_**-Si), 0.28 (Si**Me_2_**) **^29^Si NMR (80 MHz, CDCl_3_, δ, ppm)**: −80.19 (**T_4_ cycle**), 10.30 (**Si**Me_2_) **IR (ν, cm^−1^):** 3056 (C_Napht_-H), 1600 (C_Napht_-C_Napht_), 1043 (Si-O-Si), 841(Si-Me) **HRESI-MS (M + H): obtained** 1201.41 m/z expected 1201.40 m/z.

#### 3.2.4. Synthesis of Tetrakis(dimethyl(2-naphthylethyl)silyloxy)tetra(ethyl(ethanethioate))cyclotetrasiloxane (**J2**)

In a flame-dried Schlenk flask, **J1** (0.5 mmol, 600 mg, 1 eq) was dissolved in dry toluene (3.6 mL) under an inert atmosphere. AIBN (0.1 mmol, 16 mg, 5 mol%/vinyl) was added and the reaction mixture was heated to 40 °C. Once this temperature was reached, thioacetic acid (3.0 mmol, 215 µL, 6 eq) was injected and the temperature was further increased to 60 °C and left reacting overnight. The reaction mixture was evaporated under vacuum and the crude product was purified via silica column chromatography with a gradient of cyclohexane/ethyl acetate eluent (up to 10%). The solvent was evaporated under reduced pressure and the **J2** product was recovered as a colorless oil. **Yield:** 42 % (C_72_H_96_O_12_S_4_Si_8_, 316.2 mg, 0.21 mmol) **^1^H NMR (400 MHz, CDCl_3_, δ, ppm)**: 7.71 (m, 3H, **H_Napht_**), 7.55 (s, 1H, **H_Napht_**), 7.39 (m, 2H, **H_Napht_**), 7.25 (dd, 1H, J_1_ = 8.5 Hz, J_2_ = 1.5 Hz, **H_Napht_**), 2.96 (t, 2H, J = 8.5 Hz, S-**CH_2_**-CH_2_-Si), 2.79 (t, 2H, J = 8.5 Hz, Napht-**CH_2_**-CH_2_-Si), 2.29 (s, 3H, S-(C=O)-**CH_3_**), 1.05 (m, 4H, J = 8.5 Hz, Napht-CH_2_-**CH_2_**-Si and S-CH_2_-**CH_2_**-Si)), 0.20 (s, 6H, Si**Me_2_**) **^13^C NMR (100 MHz, CDCl_3_, δ, ppm)**: 195.66 (S-(**C**=O)), 142.16 (**C_Napht_**), 133.68 (**C_Napht_**), 131.94 (**C_Napht_**), 127.88 (**C_Napht_**), 127.62 (**C_Napht_**), 127.47 (**C_Napht_**), 127.03 (**C_Napht_**), 125.82 (**C_Napht_**), 125.48 (**C_Napht_**), 125.00 (**C_Napht_**), 30.69 (S-(C=O)-**CH_3_**), 29.43 (Napht-**CH_2_**-CH_2_-Si), 24.37 (**CH_2_**-S-(C=O)), 19.77 (Napht-CH_2_-**CH_2_**-Si), 15.34 (Si-**CH_2_**-CH_2_-S), 0.33 (Si**Me_2_**) **^29^Si NMR (80 MHz, CDCl_3_, δ, ppm)**: −71.58 (**T_4_ cycle**), 10.53 (**Si**Me_2_) **IR (ν, cm^−1^)**: 3055 (C_Napht_-H), 1687 (C=O), 1600 (C_Napht_-C_Napht_), 1053 (Si-O-Si), 842 (Si-Me), 624 ((C=O)-S) **HRESI-MS (M + Na)**: obtained 1527.42 m/z, expected 1527.38 m/z.

#### 3.2.5. Synthesis of Tetrakis(dimethyl(2-naphthylethyl)silyloxy)-tetramercaptoethyl-cyclotetrasiloxane (**J3**)

The THF used in this reaction was dried and outgassed with three freeze/pump/thaw cycles prior to use. In a first flame-dried Schlenk flask, a solution of LiAlH_4_ (0.42 mmol, 16 mg, 4 eq) in THF (3.2 mL) was prepared under an argon atmosphere. In another flame-dried Schlenk flask, **J2** (106 µmol, 160 mg, 1 eq) was dissolved in THF (8.0 mL). The second Schlenk flask was placed in an ice bath at 0 °C, and then the LiAlH_4_ solution was slowly added to the solution of **J2**. The reacting mixture was kept at 0 °C for 1 h and then returned to room temperature for 1 h. The reaction was neutralized with a HCl solution at pH=6 and DCM was added. The solvent was evaporated, and the crude product was re-dissolved in DCM to wash the organic phase with water. After drying over Na_2_SO_4_, the solvent was evaporated under reduced pressure. The product was purified by column chromatography with ethyl acetate/cyclohexane with a gradient of 10/90 and the product was recovered as an opaque oil. **Yield:** 28 % (C_64_H_88_O_8_S_4_Si_8_, 40.1 mg, 30.0 µmol) **^1^H NMR (400 MHz, CDCl_3_, δ, ppm):** 7.68 (m, 3H, **H_Napht_**), 7.54 (s, 1H, **H_Napht_**), 7.39 (m, 2H, **H_Napht_**), 7.25 (m, 1H, **H_Napht_**), 2.76 (t, 2H, J = 8.5 Hz, Napht-**CH_2_**-CH_2_-Si), 2.63 (m, 2H, S-**CH_2_**-CH_2_-Si), 1.59 (t, 1H, S**H**), 1.03 (m, 4H, J = 8.5 Hz, Napht-CH_2_-**CH_2_**-Si and S-CH_2_-**CH_2_**-Si), 0.17 (s, 6H, Si**Me_2_**) **^13^C NMR (100 MHz, CDCl_3_, δ, ppm):** 141.88 (**C_Napht_**), 133.61 (**C_Napht_**), 131.90 (**C_Napht_**), 127.87 (**C_Napht_**), 127.56 (**C_Napht_**), 127.36 (**C_Napht_**), 126.83 (**C_Napht_**), 125.84 (**C_Napht_**), 125.38 (**C_Napht_**), 125.01 (**C_Napht_**), 29.35 (Napht-**CH_2_**-CH_2_-Si), 20.31 (Si-**CH_2_**-CH_2_-S), 19.72 (Napht-CH_2_-**CH_2_**-Si), 19.57 (**C**H_2_-SH), 0.27 (Si**Me_2_**) **^29^Si NMR (80 MHz, CDCl_3_, δ, ppm)**: −71.87 (**T_4_ cycle**), 10.42 (**Si**Me_2_) **IR (ν, cm^−1^)**: 3051 (C_Napht_-H), 2570 (SH), 1600 (C_Napht_-C_Napht_), 1047 (Si-O-Si), 842 (Si-Me) **HRESI-MS (M + NH_4_)**: obtained 1354.38 m/z, expected 1354.39 m/z.

### 3.3. Characterization Methods

Liquid ^1^H, ^13^C and ^29^Si NMR spectra were obtained on a Bruker Advance 400 MHz spectrometer in CDCl_3_ at 25 °C and at concentrations of around 10 mg/mL. ^29^Si and ^13^C spectra were proton decoupled. High-resolution TOF-ESI mass spectra were obtained using a Waters Synapt G2-S spectrometer. FTIR spectra were measured on a Perkin Elmer Spectrum 100 apparatus equipped with a Gladia attenuated total reflectance (ATR) accessory. The spectrum of chlorodimethyl(2-naphthylethyl)silane was not obtained due to the corrosive characteristics of the compound, and the possibility of damaging the apparatus. 

## 4. Conclusions

Herein, we describe a new strategy for synthesizing all-*cis* bifunctional Janus cyclotetrasiloxanes, in which the orthogonal silsesquioxane and organic faces are independently functionalized. In a first step, an all-*cis* tetravinylcyclotetrasilanolate was modified on the silanolate face via condensation with a functional chlorosilane. In addition to introducing a functional moiety on all four sites on the silanolate face, this step significantly enhances the solubility of the resulting T_4_ molecule, facilitating the use of conventional organic synthesis approaches for modifying the vinyl site on the Si-C face. A thiol-ene click reaction was subsequently used to graft an alkylthiol moiety onto the Si-C face, thus generating an all-*cis* Janus tetrapod. To the best of our knowledge, this is the first report of a Janus T_4_ silsesquioxane bearing reactive thiol ligands on one face of the molecule.

This synthetic strategy enables a variety of all-*cis* bifunctional Janus cyclotetrasiloxanes to be envisaged, with the silsesquioxane and Si-C faces being independently functionalized. In particular, the availability of a wide range of vinyl-functionalized moieties provides access to a correspondingly broad palette of chlorosilanes, which can be used to introduce different functional groups on four corners of the silsesquioxane face. This feature, together with the proximity of the different organic groups, opens up a range of potential applications for this interesting family of molecular precursors in such areas as imaging, self-organization and sensing. The investigation of these properties, together with the synthesis of new Janus-type T_4_ tetrapod architectures, will be explored in future studies.

## Data Availability

The data presented in this study supporting the results are available in the main text and Supplementary Material. Additional data are available upon reasonable request from the corresponding author.

## Supplementary Materials

The following supporting information can be downloaded at: https://www.mdpi.com/article/10.3390/molecules27227680/s1, Figure S1: ^1^H NMR spectrum of the purified ethoxydimethyl(2-(naphthyl)ethyl)silane (CDCl_3_); Figure S2: ^13^C NMR spectrum of the purified ethoxydimethyl(2-(naphthyl)ethyl)silane (CDCl_3_); Figure S3: ^29^Si NMR spectrum of the purified ethoxydimethyl(2-(naphthyl)ethyl)silane (CDCl_3_); Figure S4: ^1^H NMR spectrum of **S1** (CDCl_3_); Figure S5: ^13^C NMR spectrum of **S1** (CDCl_3_); Figure S6: ^29^Si NMR spectrum of **S1** (CDCl_3_); Figure S7: ^1^H NMR spectrum of **J1** (CDCl_3_); Figure S8: FTIR spectra of **J1** (dark blue) **J2** (light blue) and **J3** (green); Figure S9: ^1^H NMR spectrum of **J2** (CDCl_3_); Figure S10: ^1^H NMR spectrum of **J3** (CDCl_3_); Figure S11: ^13^C NMR spectrum of **J3** (CDCl_3_).
