# Supplementary material for: Thiolated Janus Silsesquioxane Tetrapod: New Precursors for Functional Materials
Source: Molecules. 2022 Nov 8;27(22):7680. doi: 10.3390/molecules27227680 (PMC9696078; doi:10.3390/molecules27227680)
Supplement: Supplementary file 1 [file molecules-27-07680-s001.zip › molecules-2000443-supplementary.pdf]

Thiolated Janus silsesquioxane tetrapod: new precursors for functional materials

Mathilde Laird [1,*], Carole Carcel [1], Masafumi Unno [2], John R. Bartlett [3,*] and Michel Wong Chi Man [1,*]

[1] ICGM, Univ Montpellier, CNRS, ENSCM, 34293 Montpellier, France
[2] Department of Chemistry and Chemical Biology, Graduate School of Science and Technology, Gunma University, Kiryu 376-8515, Gunma, Japan
[3] Western Sydney University, Locked Bag 1797 Penrith NSW 2751 Australia

* Correspondence: mathilde.llp@gmail.com (ML); j.bartlett@westernsydney.edu.au (JB); michel.wong-chi-man@umontpellier.fr (MWCM)

Supporting information

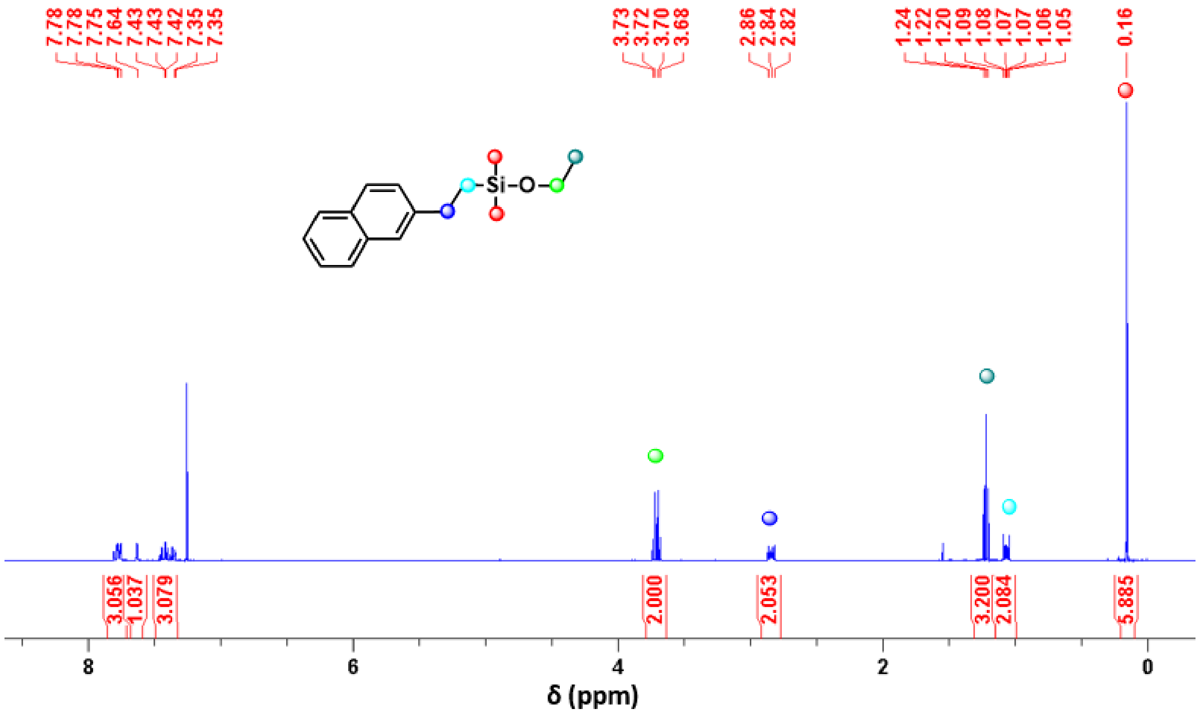

Figure S1. ^{1}H NMR spectrum of the purified ethoxydimethyl(2-(naphthyl)ethyl)silane ($CDCl_3$).

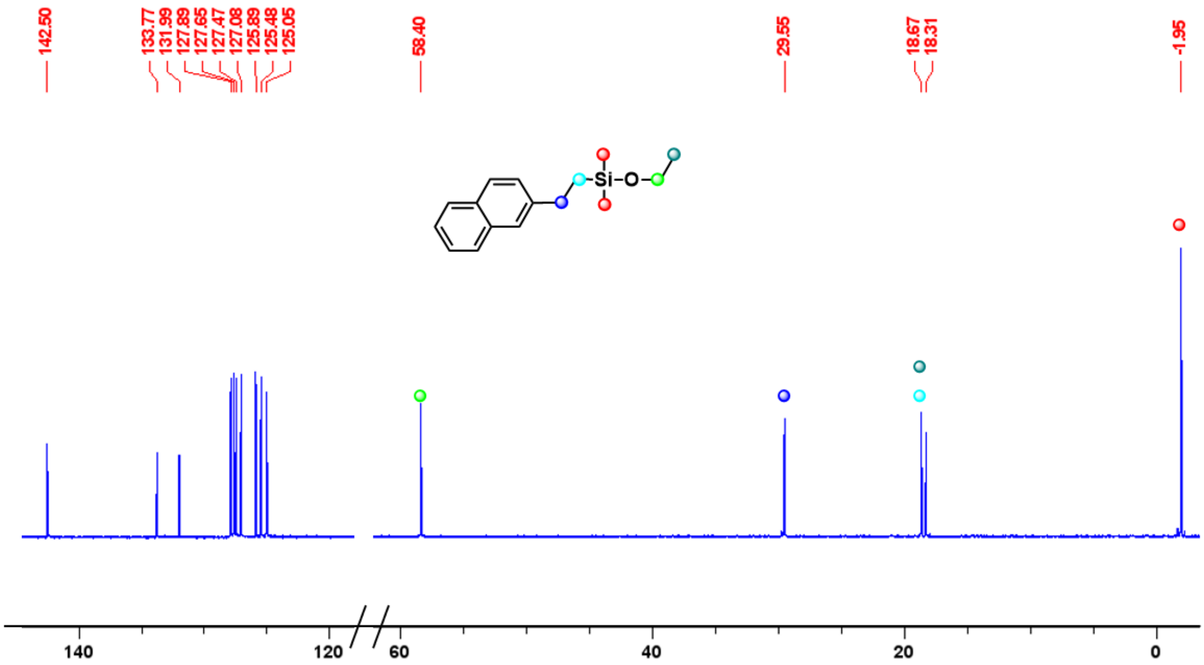

Figure S2. ^{13}C NMR spectrum of the purified ethoxydimethyl(2-(naphthyl)ethyl)silane ($CDCl_3$).

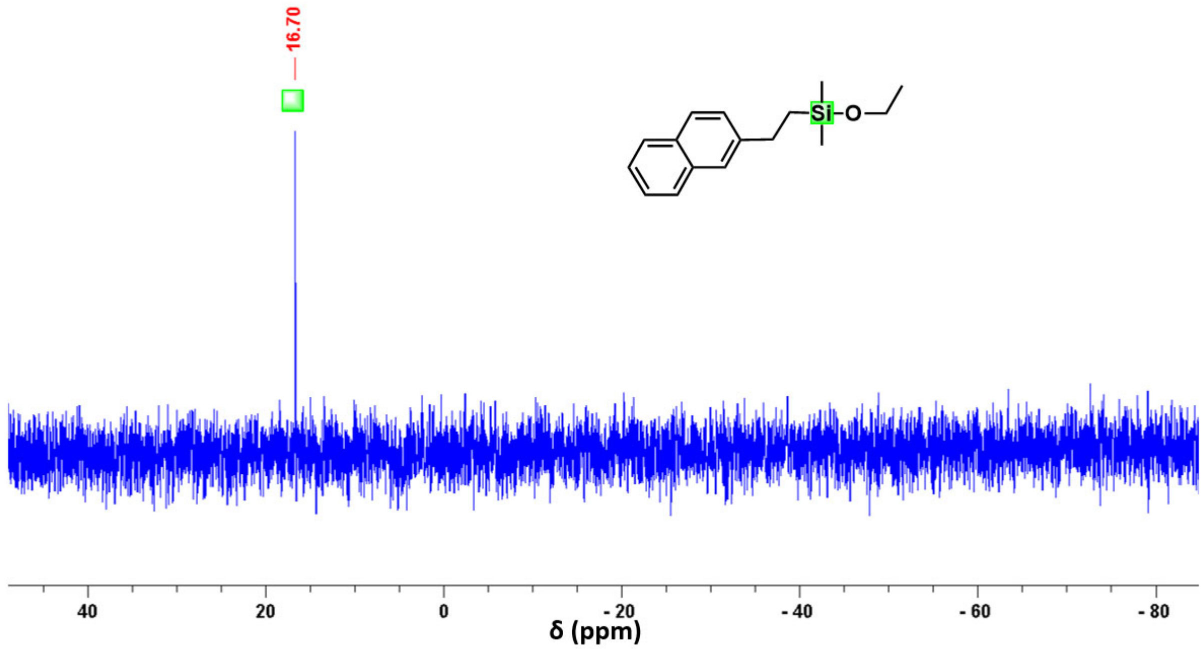

Figure S3. ^{29}Si NMR spectrum of the purified ethoxydimethyl(2-(naphthyl)ethyl)silane ($CDCl_3$).

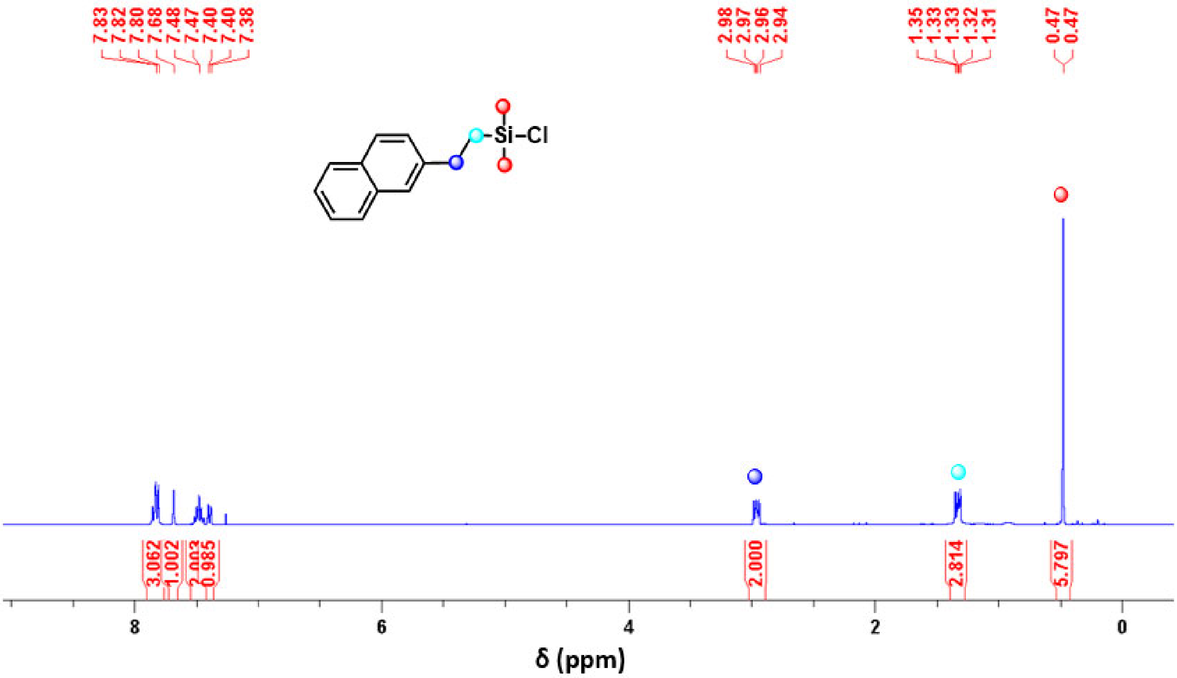

Figure S4. ^{1}H NMR spectrum of **S1** (CDCl3).

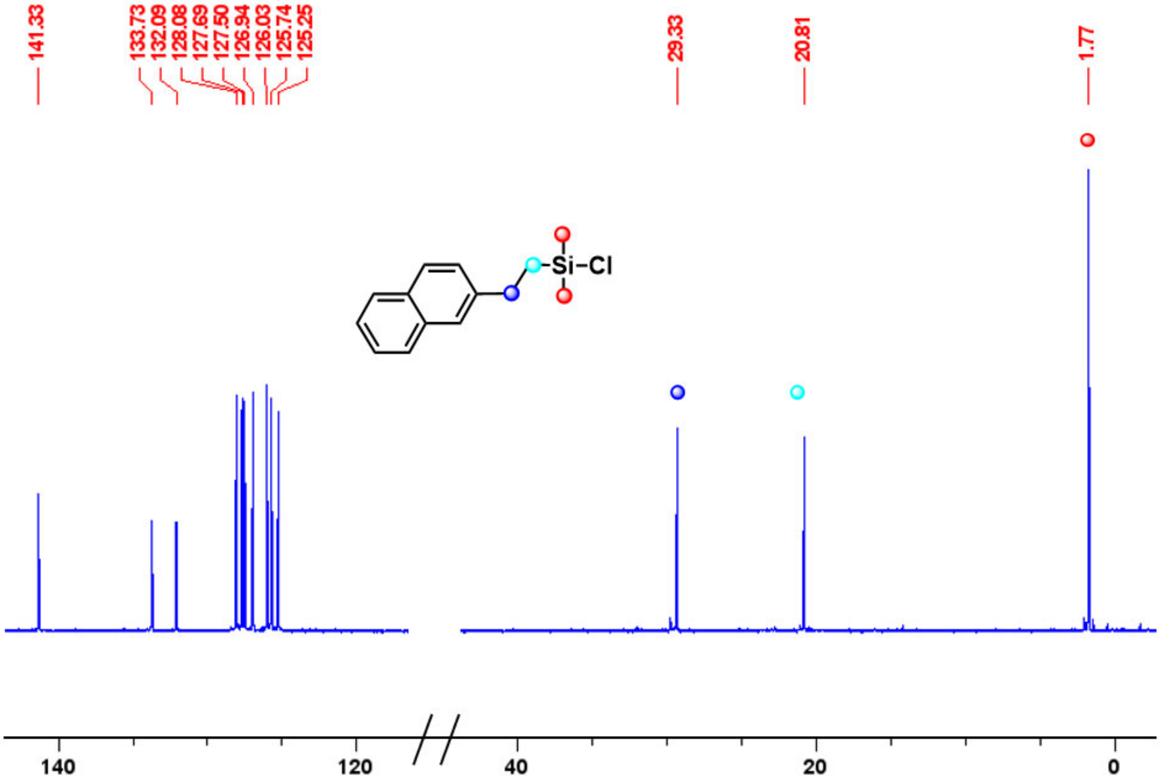

Figure S5. ^{13}C NMR spectrum of **S1** (CDCl3).

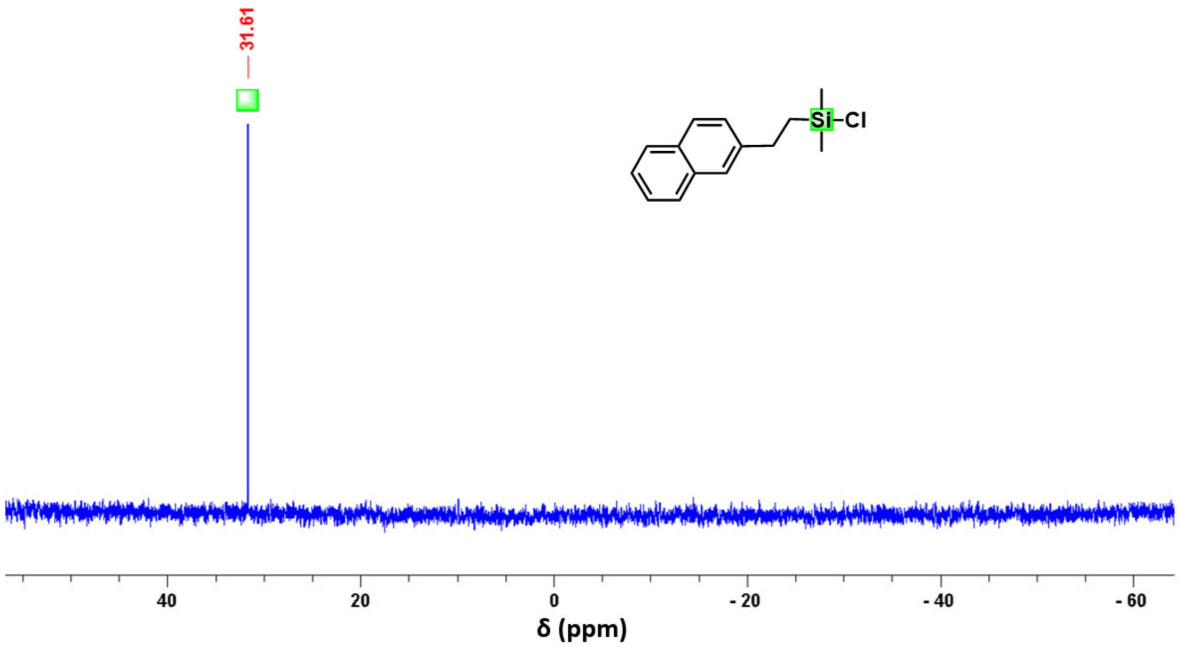

Figure S6. ^{29}Si NMR spectrum of **S1** ($CDCl_3$).

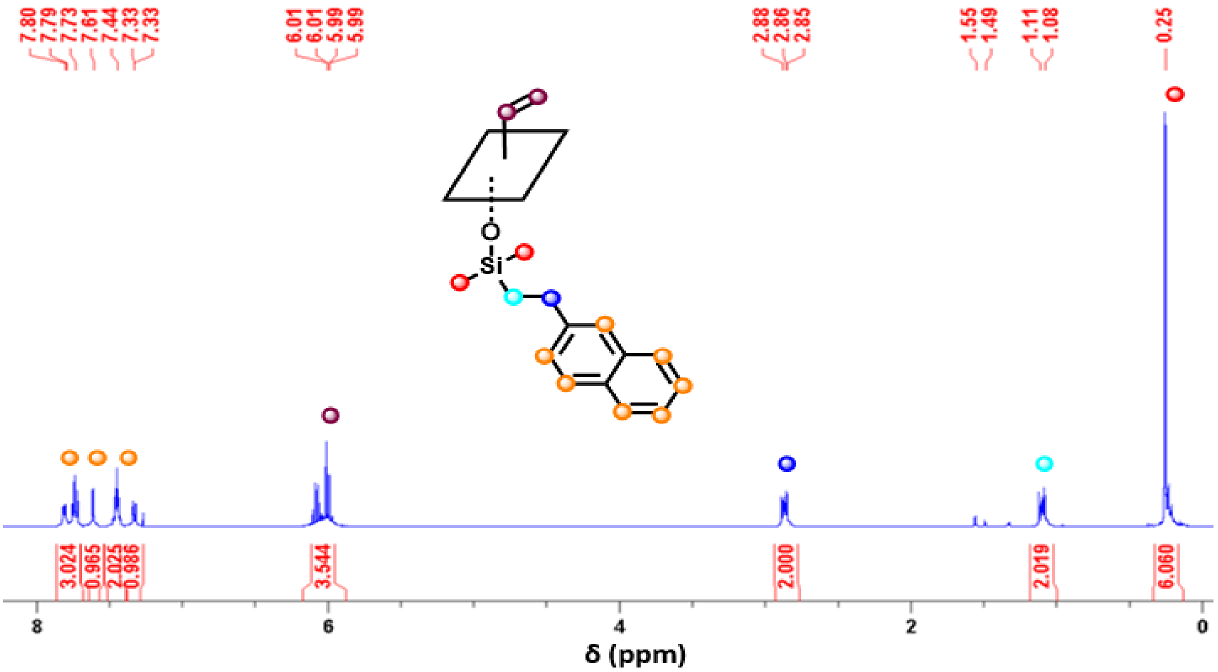

Figure S7. ^{1}H NMR spectrum of **J1** ($CDCl_3$).

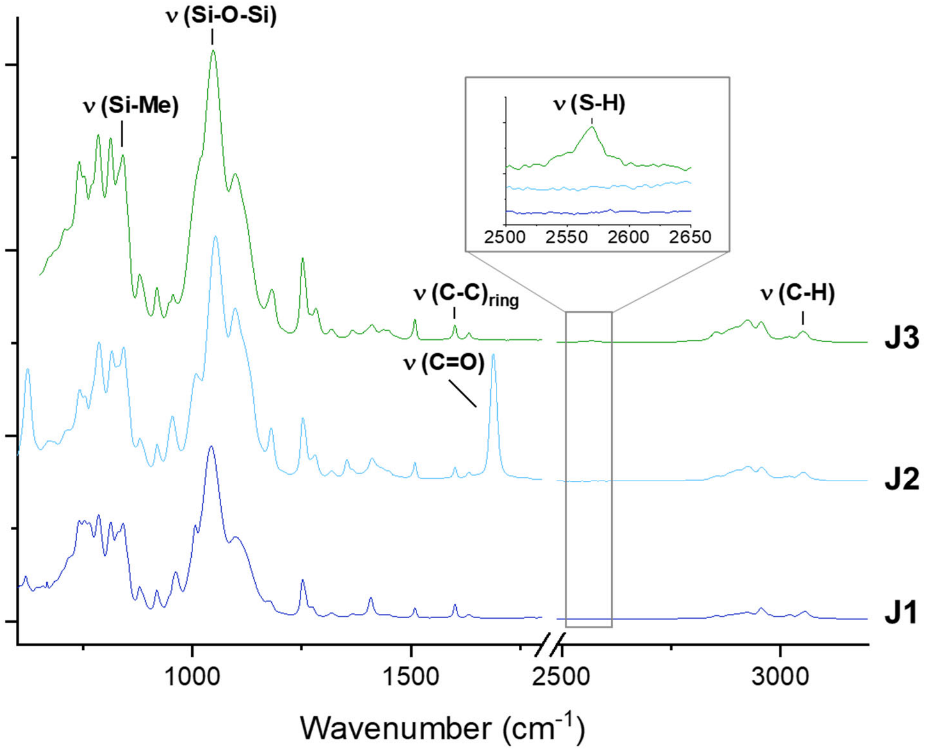

Figure S8. FTIR spectra of **J1** (dark blue) **J2** (light blue) and **J3** (green).

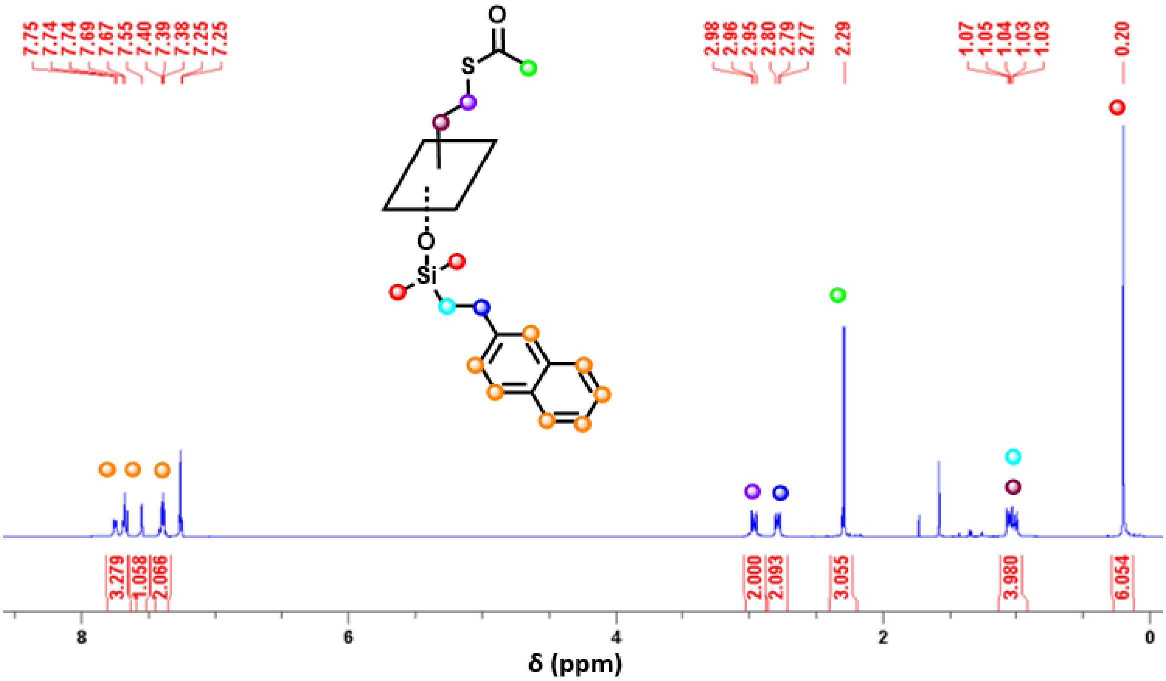

Figure S9. ^{1}H NMR spectrum of **J2** ($CDCl_3$).

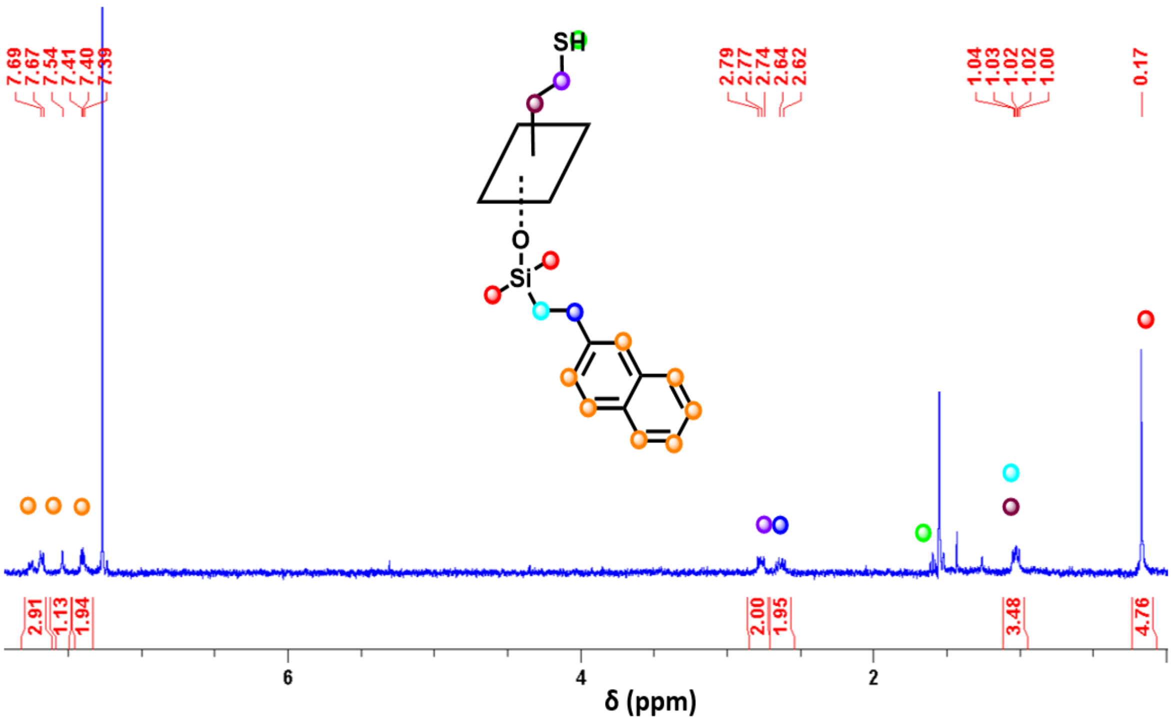

Figure S10. 1H NMR spectrum of **J3** ($CDCl_3$).

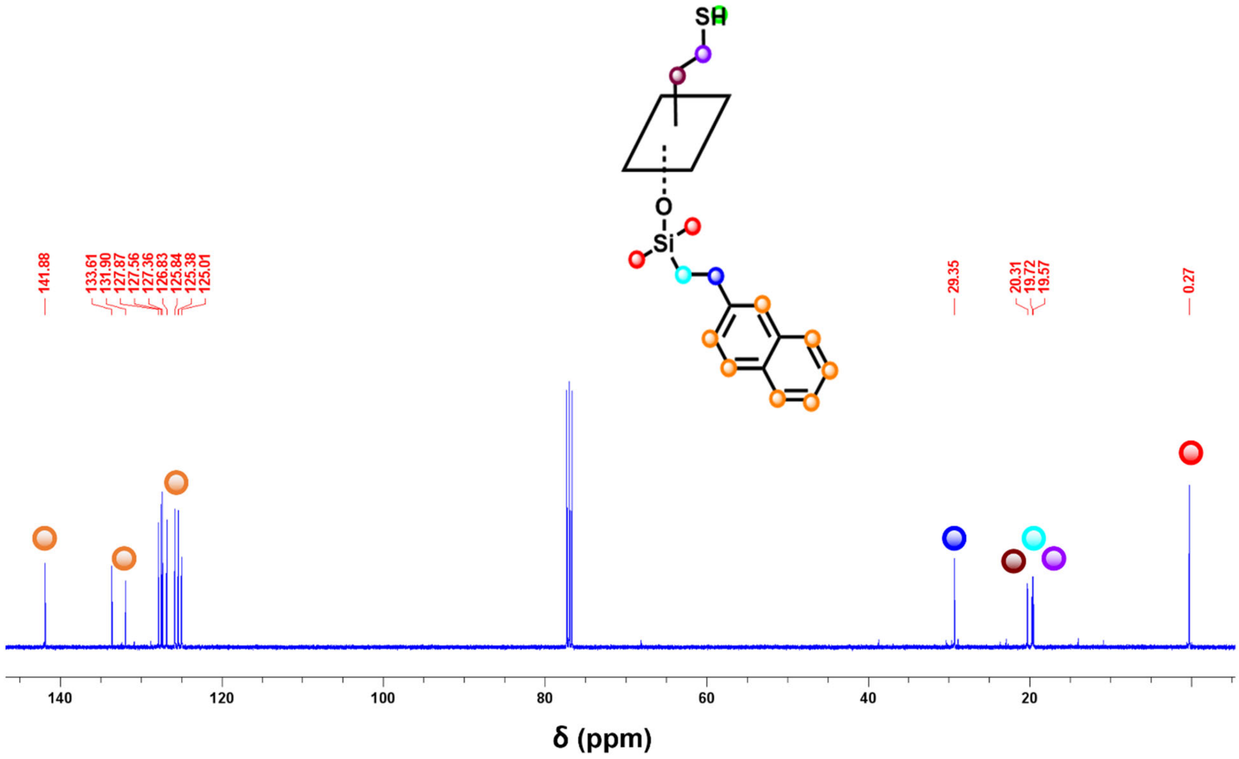

Figure S11. 13C NMR spectrum of **J3** ($CDCl_3$).
